# Associated Clinical Disorders Diagnosed by Medical Specialists in 188 *FMR1* Premutation Carriers Found in the Last 25 Years in the Spanish Basque Country: A Retrospective Study

**DOI:** 10.3390/genes7100090

**Published:** 2016-10-21

**Authors:** Sonia Merino, Nekane Ibarluzea, Hiart Maortua, Begoña Prieto, Idoia Rouco, Maria-Asunción López-Aríztegui, Maria-Isabel Tejada

**Affiliations:** 1 Molecular Genetics Laboratory, Genetics Service, BioCruces Health Research Institute, Cruces University Hospital, 48903-Barakaldo-Bizkaia, Spain; s.merino.fernandez@osakidetza.eus (S.M.); nekane.ibarluzeaguenaga@osakidetza.eus (N.I.); hiart.maortuaolabe@osakidetza.eus (H.M.); 2 Clinical Group Affiliated with the Centre for Biomedical Research on Rare Diseases (CIBERER), 46010-Valencia, Spain; malopezariztegui@yahoo.es; 3 Human Reproduction Unit, Obstetrics and Gynecology Department, BioCruces Health Research Institute, Cruces University Hospital, 48903-Barakaldo-Bizkaia, Spain; mariabegona.prietomolano@osakidetza.eus; 4 Ataxias and Spastic Paraplegias Unit, Neurology Department, BioCruces Health Research Institute, Cruces University Hospital, 48903-Barakaldo-Bizkaia, Spain; idoia.roucoaxpe@osakidetza.eus; 5 Genetics Counseling, Genetics Service, BioCruces Health Research Institute, Cruces University Hospital, 48903-Barakaldo-Bizkaia, Spain

**Keywords:** fragile X, *FMR1*premutation, FXTAS, FXPOI, hypothyroidism

## Abstract

Fragile X-associated tremor/ataxia syndrome (FXTAS) and fragile X-associated primary ovarian insufficiency (FXPOI) are definitely related to the fragile X mental retardation 1 (*FMR1*) premutation (PM). Additional medical problems have also been associated with the PM, such as fibromyalgia, endocrine, and psychiatric disorders. To improve our understanding in the field, we reviewed all PM carriers and their reasons for any medical referrals from 104 fragile X families molecularly diagnosed in our laboratory and living in the Spanish Basque Country. After signing the written informed consent, we studied their electronic medical records in order to identify the disorders associated with the PM and their frequencies. We obtained clinical data in 188 PM carriers (147 women and 41 men). In women, the frequency of FXPOI (22.61%) was similar to that previously reported in PM carriers. In men, the frequency of definite FXTAS (28.57%) was lower than reported elsewhere. Furthermore, thyroid pathology was associated with the PM, the frequency of hypothyroidism being much higher in the studied region than in the general population (8.84% vs. 0.93%). Finally, we found no association with fibromyalgia or psychiatric problems. These findings represent another population contribution in this field and may be useful for the clinical management of PM carriers.

## 1. Introduction

Fragile X syndrome (FXS) is the leading inherited cause of intellectual disability and autism spectrum disorder (ASD) [1,2,3]. The fragile X mental retardation 1 (*FMR1*) gene, discovered in 1991, encodes the fragile X mental retardation protein (FMRP), which is absent and leads to FXS when a triplet repeat expansion (CGG)—located in the 5′ untranslated region of the first exon of the *FMR1*gene—expands to more than 200 copies and the promoter region becomes hypermethylated (full mutation or FM). Individuals with alleles that range from 55 to 200 repeats are considered *FMR1* premutation (PM) carriers because women with alleles in this range may expand to more than 200 copies in the next generation [4].

Individuals with PM do not have FXS. The alleles in this range produce slightly lower levels of FMRP, but enough for patients not to develop the FXS phenotype. Therefore, PMs were only associated with a risk of expansion in the following generations. In the last 10–15 years, however, studies in these subjects have found health problems related to their status as PM carriers [2,5]. Among the molecular mechanisms underlying the fact that some of the carriers have clinical signs and symptoms, the first theory postulated was that of the mRNA toxicity due to an increase in FMR1 transcription [6,7]. Currently, other mechanisms have been proposed (reviewed in Hagerman and Hagerman, 2015) [8], such as the production of multiple antisense *FMR1* transcripts, the generation of “toxic” peptides produced by initiating at non-AUG codons located upstream of the CGG-repeat element, and the formation of R-loops both at the endogenous *FMR1* locus and in an inducible episomal system in which the expanded CGG-repeat (~95 CGG) is located [8].

There are two well-characterized disorders associated with being a PM carrier: fragile X-associated premature ovarian insufficiency (FXPOI) and fragile X-associated tremor/ataxia syndrome (FXTAS). It has been estimated that approximately 20% of women PM carriers develop hypergonadotropic hypogonadism before 40 years of age, in contrast with a rate of about 1% of the general population (reviewed in [9]). It has been observed that women at the highest risk are those with 70 to 90 CGG triplet repeats [7,9].

Fifty per cent of PM men carriers and up to 16% of PM women carriers over 50 years old develop symptoms of FXTAS [10,11,12]. This neurodegenerative disorder was discovered more than 10 years ago and is characterized by action tremor, cerebellar ataxia, and cerebral atrophy. It may also include other phenotypic features such as peripheral neuropathy and cognitive decline including executive function deficits and dementia [13,14,15]. Magnetic resonance imaging (MRI) findings have shown different signs associated with, but not limited to FXTAS (reviewed in [16]). For example, white matter hyperintensities have been observed in the middle cerebellar peduncle (MCP) in up to 58% of men and up to 13% of women with FXTAS [17], and thinning of the corpus callosum has also been observed in the majority of FXTAS patients [18]. In summary, these studies have revealed a wide and varied symptomatology similar to other neurological disorders, making a FXTAS diagnosis complex, likely resulting in under- or misdiagnosis.

In addition to FXTAS and FXPOI, numerous publications have suggested a wide variety of medical problems associated with the PM, though the relationships are not completely clear. These include dysautonomia (orthostatic hypotension, sphincter control abnormalities), psychiatric conditions (depression, anxiety), central nervous system abnormalities, sleep apnea, hypertension, rheumatologic conditions (muscle pain and/or fibromyalgia), and endocrine system diseases (thyroid disorders, among others). With regard to this topic, there are more studies in women than in men and children PM carriers [11,19].

In order to improve our understanding in the field, we undertook this work to determine how many PM carriers we studied in our region, the reasons for any medical referrals, and the frequency of clinical pathologies associated with the PM. Our hospital is the reference center for the *FMR1* gene study in the Spanish Basque Country, an autonomous region with a population of around 2,000,000. In this region, there is an independent public health system that covers 99% of the population and has an electronic health record system that provides reliable and objective information about all the patients.

## 2. Materials and Methods

We retrospectively studied and molecularly diagnosed 104 fragile X families living in the Basque Country in our laboratory (Cruces University Hospital, Barakaldo, Basque Country, Spain) during the time period from the discovery of the *FMR1* gene to 31 December 2015. We define a fragile X family when the proband carries either a FM or a PM and when at least two generations are studied.

Molecular studies were performed during these years by means of genomic Southern blot [3] or oligo Southern blot [20]. To obtain the exact number of repeats and therefore to know accurately the number of individuals molecularly diagnosed with the PM (55 to 199 CGG repeats), we reanalyzed all of the DNA samples stored with the commercial kit AmplideX^®^*FMR1* PCR (Asuragen, Austin, TX, USA) following their protocol. This enabled us to know that we have diagnosed exactly 205 PM carriers: 161 were women (78.54%), and 44 were men (21.46%). The clinical reasons for the molecular testing of the 205 PM carriers were the following: 44 were mothers of children with FXS; 5 were probands for FXTAS; 5 were probands for FXPOI; 6 PMs were found in children probands tested due to global developmental delay; 133 were relatives studied in cascade testing from a proband; 4 were prenatal diagnosis in pregnant carriers; 8 were tested for other reasons.

For the retrospective clinical study, we sent an informed consent form by post to all the PM carriers asking them to sign and return it if they agreed to us accessing their electronic medical records (Osabide/Global Clinic), which is a system of complete and unified clinical information for each patient of the Basque public health service. The study was conducted in accordance with the Declaration of Helsinki, and the protocol was approved by Comité Etico de Investigación Clínica del Hospital Universitario Cruces (CEIC E14/58).

We created a database with data on the following variables: demographic characteristics (date of birth and sex), definition of cases as a function of the reason for the genetic study, number of CGG triplets, and clinical variables diagnosed and treated by the corresponding specialists (endocrinology, rheumatology, neurology, gynecology, and psychiatry, among others).

Data analysis included comparisons with the general population, previously published data on PM carriers, or both. With regard to the descriptive statistics, we used frequencies and percentages for categorical variables, as well as means and standard deviation for the CGG repeat number variable. Finally, we used the Fisher Test to compare frequencies.

## 3. Results

Out of the initial sample of 205 PM carriers, we were able to obtain clinical data from 188 subjects: 147 women (mean age 54.68 ± 17.89; range 18–95) and 41 men (mean age 54.68 ± 22.26; range 18–85). The mean of the CGG repeat number was 83.39 ± 21.70 (range 55–180). Table 1 summarizes the global distribution of the subjects by disorder and by sex. All disorders were diagnosed by their medical specialists.

The frequency of total thyroid dysfunction was 12.23% (23/188): a single case in a man diagnosed with hyperthyroidism and 22 women. Among women, 13 patients required treatment for hypothyroidism (13/147 = 8.84%). This frequency of hypothyroidism requiring treatment is much higher than in the general population in our region (0.93%) [21] (*p* < 0.0001). All the PM carriers requiring thyroid treatment were over 41 years of age, except for two younger women.

In relation to the women diagnosed with psychiatric disorders, 24.49% (36/147) were diagnosed with anxiety and depression and required psycho active drug treatment. Among men, 12.19% (5/41) were also diagnosed with anxiety/depression disorder.

With 50.1 being the mean age of menopause in our population [22], and following a Spanish consensus [23], women over 40 years of age were considered in order to estimate frequencies of FXPOI. Among the 19 women diagnosed with FXPOI in this range, in three of the cases, the testing was requested due to the clinical diagnosis of primary ovarian insufficiency (POI), i.e., they were probands. Hence, if we exclude these patients from the retrospective study to calculate the percentage of PM carrier women with FXPOI in our population studied, the percentage would be 19.75% (16/81). In addition, among women under 40 years of age, three were diagnosed with FXPOI; in two of them, testing had also been requested due to POI.

Regarding FXTAS, according to the clinical and imaging criteria established by Jacquemont et al., in 2003 [24] and by Hall et al., in 2014 [13] for the diagnosis of FXTAS, among the 21 men >50 years old reviewed, 28.57% met the criteria for a definite diagnosis (6/21). Another patient met the criteria for a probable diagnosis, and none fulfilled the criteria for possible FXTAS. The *FMR1* gene was tested in four of these men as part of the routine tests of patients that show clinical FXTAS features, i.e., they were probands. Therefore, even including the “probable” patient, the percentage in the retrospective study of FXTAS in PM carrier men would be 17.64% (3/17). Among the 62 women reviewed, two met the criteria for definite diagnosis, one of which was a woman aged 56 referred to the laboratory because she showed clinical FXTAS features. Another woman met the criteria for a probable diagnosis, and none fulfilled the criteria for possible FXTAS. Therefore, including the “probable” patient, the retrospective study yielded a percentage value of FXTAS in PM women carriers of 3.27% (2/61).

## 4. Discussion

Despite the increasing number of publications on diseases associated with the *FMR1*premutation, it is clear that there are differences between populations and that more data is needed in order to draw conclusions that may be useful for genetic counseling and the management of the patients and their conditions. In this line, the main goal of this study was to assess the disorders diagnosed by medical specialists in adults. Data was retrieved from electronic medical records in which all of the diagnoses are assumed to be correct, because the corresponding medical specialist had documented the diagnosis. To our knowledge, this is the first time using this approach to get clinical data; most published studies have obtained their clinical data from direct interviews or surveys with the PM carriers themselves or from measurements conducted by study investigators [11,25]. Although data has been collected in a different way and results should not be compared directly, our results contribute to the knowledge of the pathologies associated to the PM.

With regard to FXPOI, frequencies of 22.6% in total and 19.75% excluding probands observed in our study are very similar to that already published (16% to 24% [9,26]). As for the mean of the PM allele CGG repeats, if we exclude the 128 PM allele considered to be an outlier in a previous study [27], the mean would be 81.33 ± 10.68 (range 59–100), also very similar to what has been reported [9,26]. In contrast, the estimated frequency for FXTAS in PM carrier men aged above 50 is 50% [10,11,12]; in our study, if we add the cases with definite and probable diagnoses frequencies of FXTAS are 33.33% in total and 17.64% excluding probands. For women, the frequencies are also lower than previously published (up to 16% [10,11,12]): 4.83% in total and 3.27% excluding probands. The statistical analysis of these differences among the previous studies and ours has not been done because direct comparisons are not a valid approach due to the differences in the ascertainment with previously published data.

A possible under-diagnosis of FXTAS may have occurred in our study population because, in PM carriers where no clinical features of FXTAS were present, MRI was not performed. Furthermore, there were only 21 men over 50 years old in our sample. We are also aware that the absence of diagnosis in our public records could be due to the fact that some subjects with only some clinical manifestations do not seek a specialist’s help or use private medicine. However, as all PM carriers receive genetic counseling after their molecular diagnosis, they know their risks and go to the specialist as soon as they believe they may have some symptoms. Apart from genetic, epigenetic, and environmental factors [15], FXTAS has recently been associated with toxicity from certain medications [28]. Hence, we consider it important to report that our only proband of FXTAS was a woman who reported the onset of all her symptoms occurring after taking interferon.

As for thyroid dysfunction, this disorder is closely related to the water and iodine consumed; therefore, comparisons should be made inside the same population. As can be seen in the results section of this paper, the frequency of hypothyroidism requiring treatment is also much higher than in the general population in the Basque Country (0.93%) [21]. These results lead us to posit that thyroid dysfunction is another condition that could be considered related to PM, a view that was previously advanced by Coffey et al. in 2008 [10] and has been reported in a recent review by Wheeler et al. that gave a figure of 17.3% [19]. Interestingly, thyroid dysfunction affects the whole range of the PM allele CGG repeats in our series.

In subjects with psychiatric disorders, according to the 2013 Basque Health Survey [29], the frequency of symptoms of anxiety and depression have significantly worsened in women, from 18% in 2002 to 24% in 2013. In our study, 24.49% of the women required medical attention and treatment for this diagnosis, similar to the current rate in the Basque general population. Furthermore, 11 of them had a child with FXS, which has been considered a determining factor for the development of this type of psychiatric problem. Therefore, the percentage of mothers with anxiety and depression who had FXS children (30.5%) is not different from the Basque general population. Hence, we do not believe that psychiatric disorders are associated with the PM in our population, although other psychiatric symptoms, which do not fit in any specialist’s diagnostic category, have not been collected in this study.

It was previously suggested that fibromyalgia is a frequent comorbidity in women carrying the PM [11,19], although in Spain two recent studies among women with fibromyalgia have reported conflicting findings [30,31], with one study finding an increased rate in PM carriers, while a second study finding no association. In the present study, only 2.12% of patients had this clinical diagnosis—women in all cases—and this frequency corresponds to that seen in the general population in Spain where the prevalence of people above 20 years of age is 2.4%, with higher rates in women (4.2%) than in men (0.2%) [32].

In conclusion, our findings report the frequency of medical pathologies in PM carriers in the Basque region of Spain, some of which are different from that previously reported in other populations.

## Figures and Tables

**Table 1:** Distribution of premutation (PM) carriers by disorder and sex.

Disorder	Sex	No. Individuals Reviewed	Affected Individuals		
			Total No.	%	Mean CGG Repeats of the PM Allele
Thyroid dysfunction	Women	147	22	14.96%	84.91 ± 24.73 (range 55–180)
	Men	41	1	2.43%	
Psychiatric disorders	Women	147	41	27.89%	80.33 ± 18.82 (range 55–137)
	Men	41	8	19.15%	
Fibromyalgia	Women	147	4	2.72%	82.50 ± 16.94 (range 67–100)
	Men	41	0	0%	
FXPOI	Women	84 (>40 years)	19 3 < 40 years old	22.61%	83.45 ± 14.41 (range 59–128)
FXTAS	Women	62 (>50 years)	2 definite and 1 probable	3.22% 1.61%	87 ± 13.12 (range 67–101)
	Men	21 (>50 years)	6 definite and 1 probable	28.57% 4.76%	

FXPOI: fragile X-associated premature ovarian failure; FXTAS: fragile X-associated tremor/ataxia syndrome.
